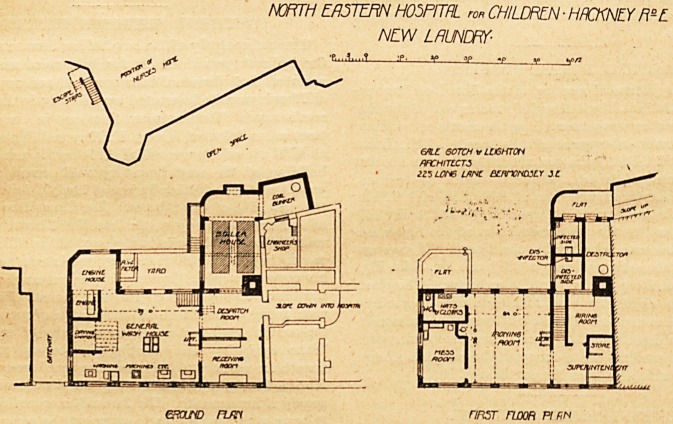# The North-Eastern Children's Hospital, Hackney Road, E.

**Published:** 1907-03-02

**Authors:** 


### THE NORTH-EASTERN CHILDREN'S HOSPITAL, HACKNEY ROAD, E.

### NEW HOME FOR NURSES AND NEW LAUNDRY.

These much-needed additions were opened by the Lord Mayor in July 1906. They will certainly prove of great advantage to the staff and to the patients, and it is further believed that they will effect a considerable financial saving on the working expenses.

### The Nurses' Home.

This is a building of oblong shape and of four stories in height. The entrance is on the centre of the west front; and the vestibule communicates immediately with a corridor five feet wide, which runs through the home from north to south, and on either side of this corridor are bedrooms. Opposite the vestibule is the main staircase, with a boot-room and store. The south-west corner of the block projects several feet beyond the line of the rest of the building, and thus provides room for the reading-room and the music-room, each of these being twenty-five feet long and fifteen feet wide, and each has a large bay window as well as other windows. There is no dining-room in the new home, as all the nurses take their meals in the hospital. The north-west and north-east angles of the block are run out into bays, one of which contains the closets (properly cut off from the block), and the other the nurses' duty-room. Close by are the bathroom and the lift, and between the bays is the fire-escape staircase. The first, second, and third floors are similar to the ground floor, but the boot-room is not carried up, and the space over the music-room and reading-room is divided into four bedrooms and a box-room. There is on the first floor a hair-shampooing room and hair-drying rooms. The nurses bedrooms are each twelve feet long, eight feet wide, and nine feet high; and the sisters' rooms are considerably larger. A special feature of this nurses' home, and a very admirable one, is that the roof is utilised as a recreation place for the staff, and this feature has been still further improved by carrying up the north-west and north-east angles to form shelters, which face the south and have glazed doors and screens. The corridors and sanitary annexes have terrazzo floors. The bedroom floors are laid down in cement, and then covered with thick linoleum. The building is therefore practically fireproof; and, considering its good means of cross-ventilation, it ought to be an eminently sanitary one. Some of the rooms are warmed by open fireplaces, and others by steam-radiators. The hot-water supply throughout is obtained by means of Royle's "Row" type of calorifier. The home will accommodate sixty nurses, and all the arrangements for comfort and safety seem good.

### The New Laundry.

The laundry adjoins the hospital, and communicates therewith. It is designed to deal with the washing for about two hundred patients and staff. It is a two-storied building. The ground floor contains receiving-room and distribution-rooms and the general washhouse, which latter is twenty-nine feet long and twenty-six feet wide. There is a drying-room and foul-linen washer. From the general wash-house a staircase leads to the first floor, which contains the ironing-room, airing-room, office, store-room, mess-room, and cloak-room. The laundry buildings are conveniently placed, and will add materially to the efficient working of the hospital. The limited area of site available for this block is, to a certain extent, responsible for the internal arrangements; but there are certain points which we consider might have been improved without altering the general outline of the building. The plan shows that the receiving-room for soiled linen can only be reached by passing through the despatch-room for clean linen. After the clothes have been dried in the drying-chamber they require to be carried back through the wash-house to the lift, which takes them up to the ironing-room. After being ironed and finished, the goods must return by the same lift into the wash-house or be carried downstairs before they reach the despatch-room. The mess-room for laundry workers should have an independent entrance, and not enter directly off the ironing-room; this point has been observed in the best types of hospital laundries, and is of the utmost importance. Ironing-rooms, more especially where gas irons are in use, require to be carefully ventilated, and should never be connected with a mess-room if this cannot be avoided, a cut-off passage with a free current of fresh air should be provided. It is not yet too late to make such a re-arrangement of the various apartments so as to obviate some of the objections to what is otherwise a very valuable addition to the hospital. The boiler-house projects towards the west side of the nurses' home, but is at sufficient distance from it, and is, of course, on a lower level than the ground floor of the block. Over the boiler-house are placed the disinfecting chambers, and there is also a "Horsfall" destructor, the flue of which runs direct into the main chimney shaft. Under the engine-house is a rain-water tank, which holds 6,000 gallons, the water being filtered before it enters the tank. In addition to this there is a Brum-Lowener patent automatic water-softener. The architects were Messrs. Gale, Gotch, and Leighton, of Bermondsey; the contractors were Messrs. Greenwood; the steel construction and staircases were carried out by Messrs. Walker, of Westminster; the sanitary fittings were by Messrs. Dent and Hellyer; the laundry machinery by Messrs. D. and J. Tullis, of London and Glasgow; the electric lifts by Messrs. Richmond and Co.; the electric lighting by Messrs. Strode and Co.; the terrazzo floors by Messrs. Diespeker. The cost of the work is not stated.

## Figures and Tables

**Figure f1:**
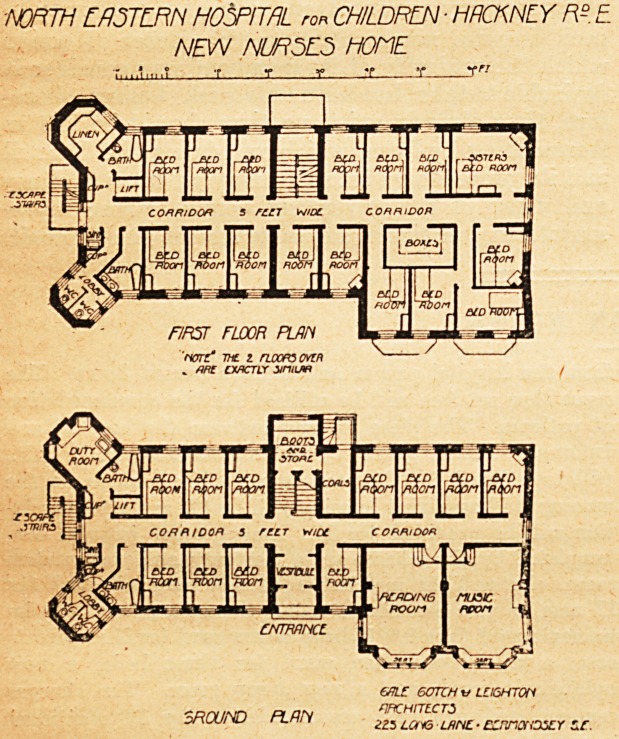


**Figure f2:**